# Stable Upconversion Nanohybrid Particles for Specific Prostate Cancer Cell Immunodetection

**DOI:** 10.1038/srep37533

**Published:** 2016-11-22

**Authors:** Yu Shi, Bingyang Shi, Arun V. Everest Dass, Yiqing Lu, Nima Sayyadi, Liisa Kautto, Robert D. Willows, Roger Chung, James Piper, Helena Nevalainen, Bradley Walsh, Dayong Jin, Nicolle H. Packer

**Affiliations:** 1 International Joint Center for Biomedical Innovation, Henan University, Kaifeng, Henan, 457001, China; 2 Department of Chemistry and Biomolecular Sciences, Macquarie University, Sydney, NSW 2109, Australia; 3 Faculty of Medicine & Health Sciences, Macquarie University, Sydney, NSW, 2109, Australia; 4 ARC Centre of Excellence for Nanoscale BioPhotonics, Macquarie University, Sydney, NSW, 2109, Australia; 5 Minomic International Ltd, Macquarie Park, Sydney, NSW, 2109, Australia; 6 Institute for Biomedical Materials and Devices, Faculty of Science, University of Technology, Sydney, NSW, 2007, Australia

## Abstract

Prostate cancer is one of the male killing diseases and early detection of prostate cancer is the key for better treatment and lower cost. However, the number of prostate cancer cells is low at the early stage, so it is very challenging to detect. In this study, we successfully designed and developed upconversion immune-nanohybrids (UINBs) with sustainable stability in a physiological environment, stable optical properties and highly specific targeting capability for early-stage prostate cancer cell detection. The developed UINBs were characterized by transmission electron microscopy (TEM), X-ray diffraction (XRD), Fourier transform infrared spectroscopy (FT-IR), dynamic light scattering (DLS) and luminescence spectroscopy. The targeting function of the biotinylated antibody nanohybrids were confirmed by immunofluorescence assay and western blot analysis. The UINB system is able to specifically detect prostate cancer cells with stable and background-free luminescent signals for highly sensitive prostate cancer cell detection. This work demonstrates a versatile strategy to develop UCNPs based sustainably stable UINBs for sensitive diseased cell detection.

Precision medicine including sensitive early-stage cancer detection holds promising potentials for lower healthcare cost and better treatment outcomes^1^,^2^,^3^. The development of cutting-edge techniques in diseased cell immunolabeling^4^, super resolution imaging^5^ and bionanomedicine^6^ has laid a good foundation and provides powerful toolboxes for advanced theranostics^7^ and the realization of precision medicine. Many commercially available bioreagents including organic dyes, chelates and fluorescent proteins have already been employed in cancer imaging and theranostics as conventional biolabels^8^,^9^. Unfortunately, their application in high sensitivity disease detection has been seriously hindered by some disadvantages, including undesirable photobleaching and photoblinking, chemical and metabolic degradation, and low signal to noise ratio^10^,^11^. These shortcomings have been partly overcome by semiconductor quantum dots (QDs)^12^, as they can possess high quantum yields, bright photoluminescence, good photostability and narrow emission, leading to their broad applications in molecular labelling as well as in cellular and *in vivo* imaging^13^. However, there have been wide concerns on the inherent toxicity, chemical instability and uncontrolled life time of QDs^14^. Furthermore, the excitation of traditional biolabels (organic dyes, fluorescent proteins, and QDs) usually requires the use of UV or short wavelength radiation for the down conversion photon transfer, which results in a series of drawbacks including low signal-to-noise ratio due to background auto fluorescence, low light-penetration depth inherent to the short wavelength of the UV excitation light, and potential cellular damage caused by long-term irradiation^15^,^16^,^17^. Therefore, it is highly desirable to produce a new class of fluorescent sensors that can label target cells or tissue with higher signal to noise ratio, stronger light penetration capabilities, better photo stability and negligible tissue photo-damage.

Upconversion nanoparticles (UCNPs) are nanoscale crystals doped with rare earth ions. They absorb in a stepwise manner two (or more) low-energy photons in near infrared (NIR) light before emitting one high-energy photon with visible luminescence^18^,^19^,^20^. Over the past decade, several studies on UCNPs have made tremendous progress, particularly in the controlled synthesis to produce mono-dispersed UCNPs with tunable nanostructure, sizes, shapes, luminescent emitting colors and life time^21^,^22^,^23^,^24^. Furthermore, many advantages of UCNPs have been recently discovered including embeddable capacity for multi-functional hybrid nanomaterials^25^,^26^, negligible cytotoxicity for compatible biomedical devices^27^, robust photo-stability for super stable diagnostics and long-term tracking of molecules and nanocarriers^28^,^29^, high bright luminescent signals with low background for super sensitive detection^30^ and deeper penetration capability for high resolution deep tissue imaging^31^,^32^. Ultrasensitivity of UCNP-based detection probes can be obtained, on one hand, by the unique property of the anti-Stokes shift which eliminates the background noise originating from the test sample. On the other hand, their long lifetime, which extends the emission period by the order of tens of microseconds, provides an opportunity for time-gated detection to remove autofluorescence and excitation scattering. All of these leads endow UCNPs the potentials to be a promising sensitive nanoprobe for early-stage cancer detection^33^. However, UCNPs are often seriously aggregated in aqueous solutions owing primarily to the presence of hydrophobic capping ligands that are used for the synthetic control of nanostructure, size and shape uniformity^24^,^30^,^34^,^35^. Moreover, a variety of targeting molecules such as proteins and peptides need to be conjugated onto the surface of UCNPs for specific cell recognition, towards super sensitive disease detection^36^,^37^.

To this end, various surface modification and functionalization strategies have been investigated to transfer such passivated nanocrystals from organic solution into aqueous solutions and to impart them targeting capability for various biomedical applications^38^. These methods, such as, capping ligands removal^39^, layer-by-layer assembly^40^, optimized salinization chemistry^41^, silica coating^42^,^43^, polymer encapsulation^44^ and ligand exchange^45^,^46^,^47^ have been found to be promising. However, most of approaches normally involve several chemical modification steps, which leads to low yield, poor stability, and low reproducibility^34^,^48^. Moreover, each surface modification will change the interface charge equilibration, which may result in instability and/or further aggregation of UCNPs^49^. Most importantly, the improper modification of antibody or other functional proteins can decrease the targeting capability of the antibody^31^,^50^,^51^,^52^. The ideal functionalization strategies should make the UNCPs stable in the physiological environment and attach the bioactive proteins without losing their targeting functions in one step. Realizing these requirements is extremely important for high sensitivity disease detection, but is also very challenging.

In this study, we successfully designed and developed upconversion immune-nanohybrids (UINBs) via one-step ligand exchange strategy. The resulted UINBs show sustainable stability in a physiological environment, super stable optical properties and highly specific targeting capability for early-stage prostate cancer cell detection. As illustrated in Fig. 1, streptavidin was initially conjugated to phosphate-poly (ethylene glycol) (PEG, 5 kDa)-carboxyl (PO_4_-PEG_5000_-COOH) via EDC chemistry and then introduced onto the surface of UCNPs together with excess PO_4_-PEG_5000_-COOH resulting in a one-step ligand exchange strategy. Finally, a highly specific antibody (MIL-38)^53^ recognizing a prostate cancer antigen was functionalized via biotinylation and introduced onto the surface of the UNCPs through the biotin-streptavidin interaction to give UINBs. The developed UINBs were characterized by transmission electron microscopy (TEM), X-ray diffraction (XRD), Fourier transform infrared spectroscopy (FT-IR), dynamic scattering light (DLS) and luminescence spectroscopy. The targeting function of the biotinylated antibody nanohybrid was confirmed by immunofluorescence assay and western blot analysis. The UINB system was able to specifically detect prostate cancer cells with stable and background-free luminescent signals for highly sensitive prostate cancer cell detection. This work demonstrates a versatile strategy to develop UCNP based sustainably stable UINBs for sensitive diseased cell detection.

## Result and Discussion

### Fabrication and Characterization of UINBs

**Figure 1:**
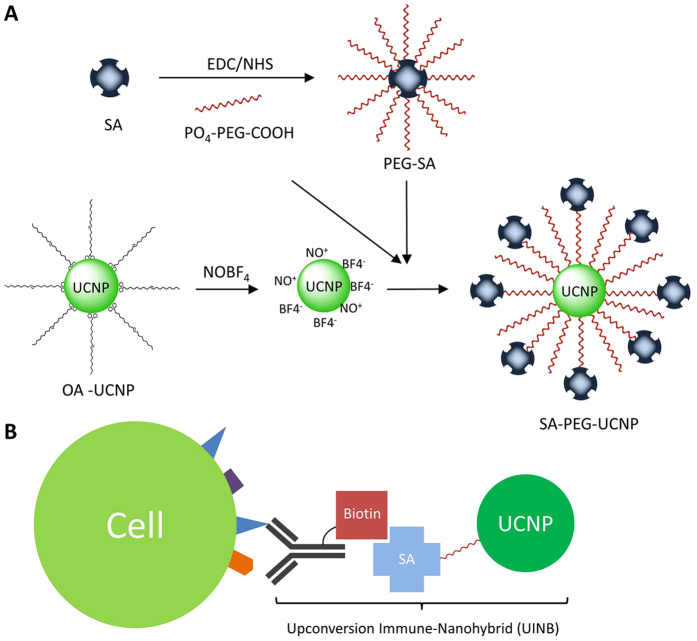
The schematic illustration of (**A**) UCNPs and PEGylated SA (PEG-SA) bioconjugation via one-step ligand exchange process. (**B**) upconversion immune-nanohybrid (UINB) driven precision cancer cell detection.

**Figure 2:**
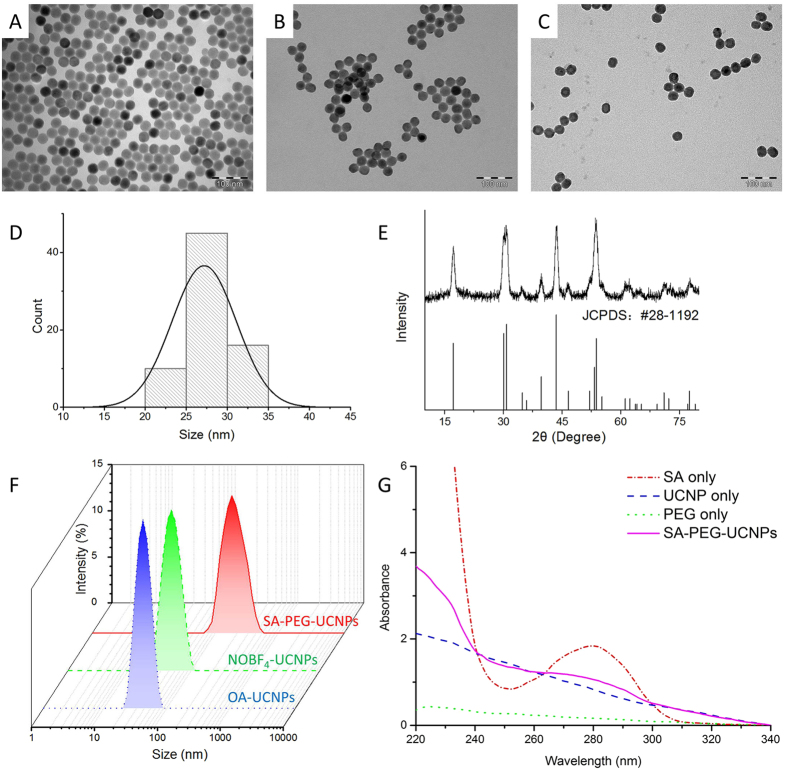
The characterizations of functionalized UCNPs: (**A**) the TEM image of naked OA-UCNPs; (**B**) the TEM image of NOBF_4_-UCNPs; (**C**) the TEM image of SA-PEG-UCNPs; (**D**) the size histogram of OA-UCNPs; (**E**) the XRD spectra of naked UCNPs; (**F**) the comparison of size distribution of OA-UCNPs, NOBF_4_-UCNPs and SA-PEG-UCNPs; (**G**) the comparison of absorbance of OA-UCNPs, NOBF_4_-UCNPs and SA-PEG-UCNPs.

UINBs were fabricated using NaYF_4_: 20% Yb^3+^/2% Er^3+^ UCNPs^54^, further functionalized with prepared PEG-Streptavidin (PEG-SA) conjugates and PEG-COOH via a one-step ligand exchange strategy (Fig. 1A). The synthesized UCNPs were originally dispersed in the organic solvent (chloroform) and kept stable with the capping agent of hydrophobic oleic acids (OA)^55^. The uniform morphology (27 ± 8.2 nm) was confirmed with TEM and DLS (Fig. 2A,D and F). Furthermore, the crystal structures and the phase purity of the OA-capped UCNPs were examined by XRD. Typical XRD patterns of the OA-capped UCNPs are presented in Fig. 2E. The diffraction peaks of the UCNPs are well defined, and the peak positions and intensities match well with the calculated values for hexagonal NaYF_4_ (β-NaYF_4_) (JCPDS No.028–1192), which indicates that the prepared UCNPs are of highly crystalline pure hexagonal phase. To transfer the hydrophobic OA-capped UCNPs into a hydrophilic intermediate for further biomedical applications, NOBF_4_ was used as an agent to replace the original OA capping and form an intermediate of NOBF_4_-UCNPs (Fig. 2B)^56^,^57^,^58^. The replacement of NOBF_4_ can be confirmed by FT-IR spectra (see details in Supplementary Information, Figure S3). During the NOBF_4_ treatment, the OA can be efficiently stripped off and inorganic BF_4_^−^ anions replaced and coordinated with lanthanide dopant ions on the surface of UCNPs and provide electrostatic stabilization in polar media. Due to the BF_4_^−^ has low binding affinity to the UCNPs surface, it can be replaced by other capping reagents such as phosphates which allows for further ligand exchange functionalization.

Streptavidin (SA) was modified with a PEG linker (PO_4_-PEG_5000_-COOH, ~5 kDa) via EDC chemistry with the formation of amide bonds between carboxylic groups of PEG linker and primary amine groups of SA. The PEG linker modification was used here to reduce the steric hindrance at the surface of the UCNP and increase the stability of the potential biotin-streptavidin interaction. A reaction ratio of 50 (molar ratio: PEG linker to SA) was chosen to keep the balance between product yield and SA biological activity because the less modification, the higher original bioactivity^59^,^60^. The SA-PEG reaction mixture was analyzed by SDS-PAGE electrophoresis as shown in Supplementary Figure S1A; high molecular weight protein bands were observed at approximately 28 kDa, 38 kDa, 58 kDa and 63 kDa in the PEGylated sample compared with the SA alone (13 kDa, in monomer phase) control, which confirmed the successful SA PEGylation and based on mass, indicated that 3, 5, 9 and 10 PEG linkers have been conjugated on one single SA monomer, respectively. The result from ImageJ analysis of the gel band intensities estimated that there is 57.6% SA modification with PEG and the main product is triply PEGylated SA monomer (50.3%) (Supplementary Figure S1B). Accordingly, 12 PEG chains were estimated to be conjugated on one SA tetramer. The PEG-SA mixtures were further purified by size exclusion chromatography to obtain the highest pure PEG-SA of the main products (112 kDa, with 12 PEG chains per SA), followed by one-step ligand exchange of UCNPs with the purified 12 PEG-SA conjugation in excess unmodified phosphate PEG (5 kDa). This approach replaces the BF_4_^−^ counter-ions on UCNPs with PO_4_^3−^ groups on either PEGylated SA or PEG to form water soluble SA-PEG-UCNPs (Fig. 2C). The excess PO_4_-PEG_5000_-COOH was applied here to coat any remaining bare UCNP surface. This process is used to reduce non-specific interaction and enhance the stability and biocompatibility of the synthesized SA-PEG-UCNPs in the biological solutions by its hydrophilic nature^59^.

The dynamic size change of the SA-PEG surface-modified UCNPs were measured by DLS. As shown in Fig. 2F, the hydrodynamic average diameters of UINBs were significantly increased from 36.68 nm (OA-capped UCNPs) to 161.8 nm (SA-PEG-UCNPs) after surface modification, which is mainly attributed to the diameter of SA and the long PEG chains. On the other hand, the zeta potential was decreased from 23.3 mV to −36.1 mV after conjugation due to the introduction of negative charged PEG molecules and SA protein (Supplementary Figure S2). The SA-PEG-UCNP conjugates can also be verified by checking the typical OD280 absorbance peak from a Nanodrop spectrophotometer (Fig. 2G). SA shows a strong ultraviolet (UV) absorption peak at 280 nm while UCNP sample does not have such a typical absorbance. After conjugation, the absorption profile of SA-PEG-UCNPs displays an increasing absorbance at 280 nm. In addition, the functional groups on the surfaces of the bare and SA-PEG-UCNPs before and after bioconjugation were characterized and confirmed by FT-IR spectra (Supplementary Information, Figure S3). The significant changes in surface properties (size, zeta potential and FT-IR spectra) indicate the success of surface functionalization with SA.

**Figure 3:**
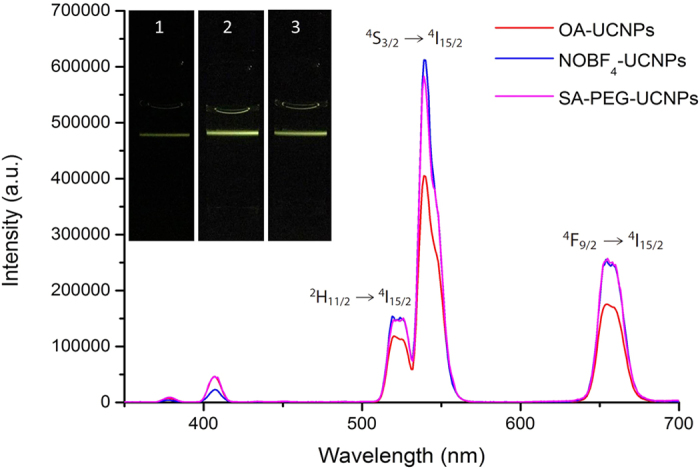
Comparison of upconversion fluorescence emission spectra between (red trace) OA-UCNPs dispersed in cyclohexane, (blue trace) NOBF_4_-UCNPs dispersed in DMF and (purple trace) SA-PEG-UCNPs dispersed in PBS buffer under 980 nm excitation. The upper panel photographs and lower spectra display the luminescence excited with a 980 nm laser of (1) OA-UCNPs, (2) NOBF_4_-UCNPs and (3) SA-PEG-UCNPs. All spectra and photographs were obtained at the same concentration of UCNPs (10 mg/mL).

To investigate the effect of the surface modification of UCNPs on the upconversion luminescence (UCL) properties, we measured the photoluminescence spectra of these UCNPs at the same concentration before and after surface modification, by exciting the dispersions in PBS buffer with a 980 nm diode laser. As shown in Fig. 3, the SA-PEG-UCNPs have good dispersibility in water after functionalization with PEG-SA, and the resulting SA-PEG-UCNPs retained the characteristic upconversion optical properties of the NOBF_4_ particles with 1.5 fold increased fluorescent intensity, compared to that of the prepared OA-capped UCNPs in cyclohexane. The two strong green emissions at 520 and 540 nm can be assigned to the ^2^H_11/2_→^4^I_15/2_ and ^4^S_3/2_→^4^I_15/2_ transitions, respectively, whereas the weak red emission at 654.5 nm can be assigned to the ^4^F_9/2_→^4^I_15/2_ transition. The upper panel of Fig. 3 shows the UC fluorescence of the colloidal solution excited with a 980 nm laser appears green in colour and the variation in fluorescence intensity confirmed the result in UCL emission spectra comparison.

### Sustainable Stability of SA-PEG-UCNPs

**Figure 4:**
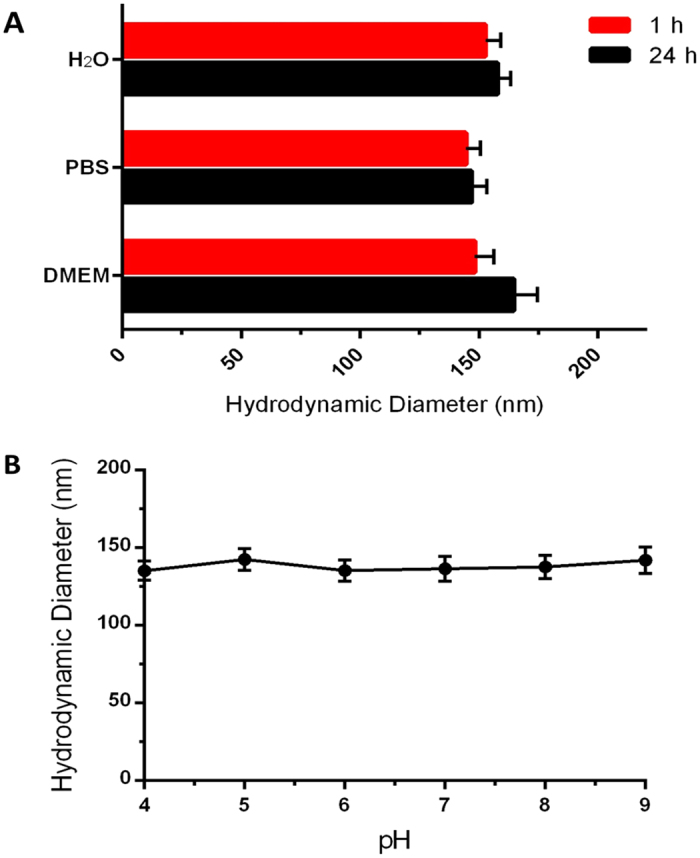
(**A**) The dynamic light scattering (DLS) of SA conjugated UCNPs dispersed in H_2_O, 0.01 M PBS buffer and DMEM cell culture medium with 10% FBS at 1 h and 24 h after preparation. (**B**) DLS of SA conjugated UCNPs dispersed in PBS buffer at different pH from 4.0 to 9.0 in 1 h.

To investigate the stability of SA-PEG-UCNPs under physiological conditions, the SA-PEG-UCNPs were dissolved at a concentration of 1 mg/mL PBS and Dulbecco’s modified Eagle’s medium (DMEM with serum) using water as control. The results from the DLS analysis demonstrate that SA-PEG-UCNPs form good colloidal dispersions, with the particle size in water and PBS similar from 1 h to 24 h later, while in DMEM the size increased slightly (from 150 nm to 165 nm) after 24 h’ incubation (Fig. 4A). The sustainable stability of SA-PEG-UCNPs in various physiological buffers was also confirmed by DLS measurement and by exciting the colloidal dispersions with a 980 nm diode laser. As shown in the left panel of Supplementary Figure S4A, strong the UCL emitted from nanoparticles in different solutions (water, PBS and DMEM) was observed with naked eyes from the top layer (left photograph) and bottom layer (right photograph) of the solution when excited with a NIR laser at the top and bottom part of vials, respectively. After 24 h dispersion of UCNPs, green colored fluorescence still can be seen from top layer of solution suggest a good water stability of SA-PEG-UCNPs under physiological conditions as aggregated nanoparticles settle to the bottom rapidly. DLS analysis of SA-PEG-UCNPs in different solutions shows that there is no obvious change of the particle sizes within 24 h (Supplementary Figure S4A). In addition, the size of SA-PEG-UCNPs were tested in PBS at various pH values (4 to 9); no significant change was observed within 1 h, suggesting stable colloidal dispersions of SA-PEG-UCNPs independent of pH (Fig. 4B, S4B). Even highly concentrated colloidal dispersions of SA-PEG-UCNPs (∼5 mg/mL) are stable for several months (data not shown) without sedimentation or precipitation as examined by DLS analysis. Such a sustainable stability of SA-PEG-UCNPs was thus achieved via a one-step ligand exchange, benefiting from the robust attachment of phosphate groups to UCNPs surfaces and the long hydrophilic PEG linker chain. The stability of these SA-PEG-UCNPs allows their application in biological systems. To test their cytotoxicity, the effect of the developed SA-PEG-UCNPs on cell viability was examined by the MTT assay. The results showed that the cell viability was above 90% over 48 h exposure to up to 200 g/mL of the conjugated UCNPs (Supplementary Figure S5), demonstrating good biocompatibility.

### Validating the Antibody-Biotin-SA Conjugation and Specificity

Another key factor for highly sensitive disease detection is the use of highly specific antibody that targets a unique biomarker of diseased cells. MIL-38 (a IgG_1_ murine monoclonal antibody) is a prostate cancer antibody^61^,^62^ detecting a cell surface glycoprotein and was supplied by our industry partner (Minomic Int. Ltd). The specific targeting capability of MIL-38 antibody was tested via a standard FITC conjugated immunofluorescence assay on a range of cell lines including one positive cell line (DU145, prostate cancer cell) and four negative cell lines (prostate cancer cell: LNCaP, and breast cancer cells: MCF-7, MDA-MB-231 and SK-BR-3). As shown in Supplementary Figure S6, FITC labelled MIL-38 antibody only targeted DU145 cells with high specificity, with no signal detected on the non-cancer or other cancer cells, indicating the excellent targeting capability of MIL-38 antibody to prostate cancer cells (DU145).

**Figure 5:**
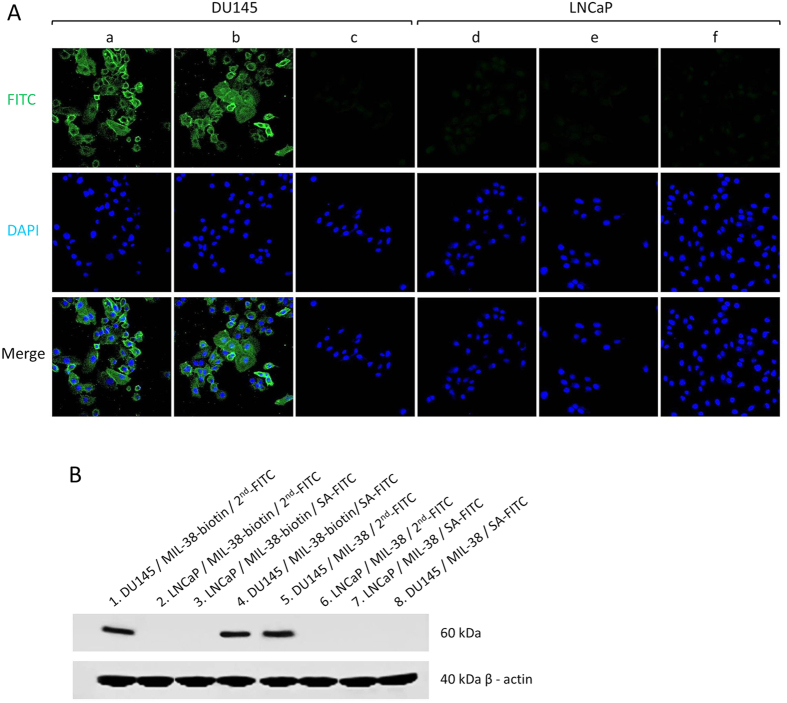
(**A**) Confocal luminescence imaging of (a) MIL-38-biotin incubated with SA conjugated FITC (SA-FITC), (b) MIL-38-biotin incubated with goat anti-mouse secondary antibody-FITC (2^nd^-FITC) and (c) MIL-38 incubated with SA-FITC on DU145 prostate cancer cells. (d) MIL-38-biotin incubated with SA-FITC, (e) MIL-38-biotin incubated with 2^nd^-FITC and (f) MIL-38 incubated with SA-FITC on LNCaP prostate cancer cells. Green and blue colors represent green and blue fluorescence from FITC and DAPI, respectively. (**B**) Western blot analysis of biotinylated antibody MIL-38-biotin incubated with 2^nd^-FITC and SA-FITC on the proteins isolated after DU145 and LNCaP cell lysis (1–4); and antibody MIL-38 incubated with 2^nd^-FITC and SA-FITC after DU145 and LNCaP cell lysis (5–8). Lower panel shows the western blot analysis with β-actin as loading control.

MIL-38 antibody was then biotinylated using EZ-link Sulfo-NHS-LC-biotin through the well-known reaction between primary amine groups on the antibody molecule and NHS activated biotin. The success of the biotinylation of MIL-38 was validated with FITC conjugated SA (SA-FITC) by immunofluorescence assay (IFA) and western blot (WB) analysis using LNCaP cells as negative control. In the IFA assay, DU145 cells (positive) and LNCaP cells (negative) were incubated with biotinylated MIL-38 (MIL-38-biotin) as a primary antibody. Subsequently SA-FITC and FITC conjugated goat anti-mouse secondary antibody (2^nd^-FITC) were applied to detect MIL-38-biotin on the cell surface. As shown in Fig. 5A(a and b), using confocal microscopy, both SA-FITC and 2^nd^-FITC labelled DU145 cells through specific SA-biotin interaction and MIL-38 antibody-goat anti-mouse secondary antibody recognition, labelled DU145 cells respectively compared to the non-biotinylated MIL-38 control (Fig. 5A c).

No signal was detected on the LNCaP cells with MIL-38 labeling under any conditions, suggesting the highly specific targeting capability of MIL-38 was maintained after biotinylation. Figure 5A(d,e and f) confirms that SA did not non-specifically bind to the cells. WB was also performed to further verify the specific targeting capability of MIL-38-biotin. In WB assay, cell lysates proteins of DU145 and LNCaP cells were gel-separated, and SA-FITC and 2^nd^-FITC were tested with the different MIL-38 conjugates, using β-actin (one common protein in both cells) as loading control. As shown in Fig. 5B, the bands at Lane 1 and 4 show the MIL-38 biotinylated antibody binding of a DU145 protein can be detected by both 2^nd^-FITC and SA-FITC. In addition, the MIL-38 antibody recognition of the DU145 antigen can be targeted by 2^nd^-FITC alone (band in Lane 5). In addition, none of the immunoblotting assays showed detection in the LNCaP control, indicating there is no MIL-38 antigen in the LNCaP cell lysates and confirms the highly specific targeting capability of MIL-38-biotin and the specific interaction between biotin and SA. These results thus indicate the successful MIL-38 biotinylation and labelling specifically of prostate cancer cells with the traditional FITC fluorophore labelling.

### Immunolabeling and Imaging of Prostate Cancer Cells with UCNPs

**Figure 6:**
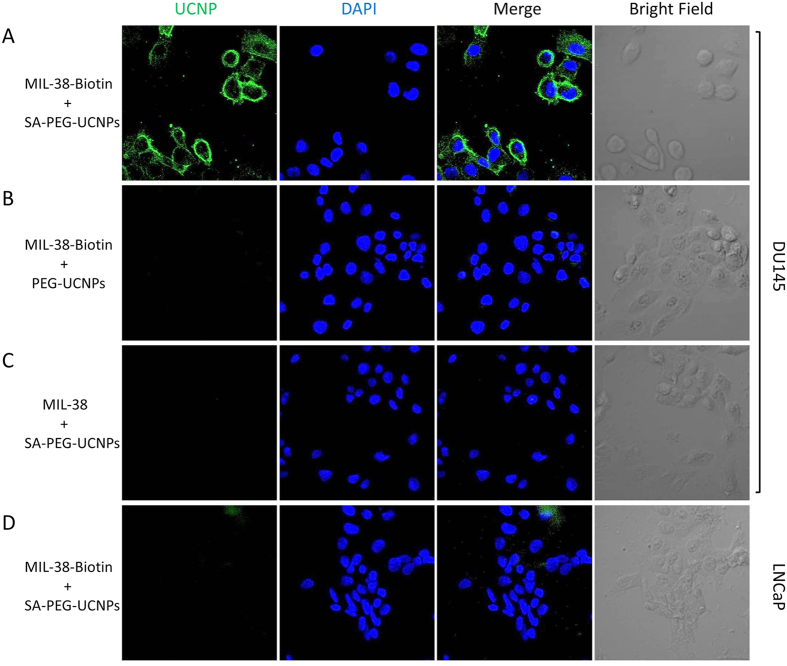
Confocal upconversion fluorescence imaging of (**A**) SA-PEG-UCNPs + biotinylated MIL-38 (MIL-38-Biotin) labelled DU145 prostate cancer cells; (**B**) PEG-UCNPs + MIL-38-Biotin labelled DU145 prostate cancer cells; (**C**) SA-PEG-UCNPs + MIL-38 labelled DU145 prostate cancer cells; (**D**) SA-PEG-UCNPs + MIL-38-Biotin labeled LNCaP prostate cancer cells. Green and blue colors represent upconversion fluorescence signals and blue fluorescence from UCNPs and DAPI, respectively.

To facilitate the detection of prostate cancer cells with UINBs, MIL-38-biotinylated antibody was linked to streptavidin coated UCNPs (SA-PEG-UCNPs) as shown in Fig. 1B. The resulting MIL38-biotin-SA-PEG-UCNPs were incubated with prostate cancer cells (DU145) in physiological conditions (0.01 M, pH 8.0 PBS buffer in room temperature), with LNCaP cells as negative control. The highly specific immunoreaction between the MIL38-biotin-SA-PEG-UCNPs and cell surface protein expressed on DU145 prostate cancer cell membranes was seen. The cells were washed thrice after incubation with MIL38-SA-PEG-UCNPs and images were captured using a confocal microscope equipped with a 980 nm NIR laser. It can be clearly seen in Fig. 6A that the DU145 cells exhibited bright green UC fluorescence on their membranes. The shape and position of the cells in bright field and dark field overlapped very well, showing good luminescent signal for detection and imaging. No UC fluorescence was detected on LNCaP cells under the same conditions (Fig. 6D), confirming the specific labelling of the antibody-UCNP conjugates to the cancer specific surface antigens on prostate cancer cells.

As controls for the specificity of the developed UNIBs, MIL38 antibody-biotin + PEG-UCNPs mixtures and MIL38 antibody + SA-PEG-UCNPs mixtures were incubated with BSA-blocked DU145 cells (positive) and LNCap cells (negative) under the same conditions and detected with a 980 nm NIR laser equipped confocal microscope. Binding of MIL-38 antibody was not seen without SA (Fig. 6B) or biotin (Fig. 6C) on DU145 cells or on LNCaP cells (Supplementary Figure S7C & D). It is well-known that the autofluorescence (noise) from cells is very low in the UCNPs based systems due to the unique UC mechanism of the NIR light excited UCNPs, which results in a higher signal-to-noise ratio for bioimaging^34^,^52^,^63^.

### Optical Stability for Precision Cancer Detection

**Figure 7:**
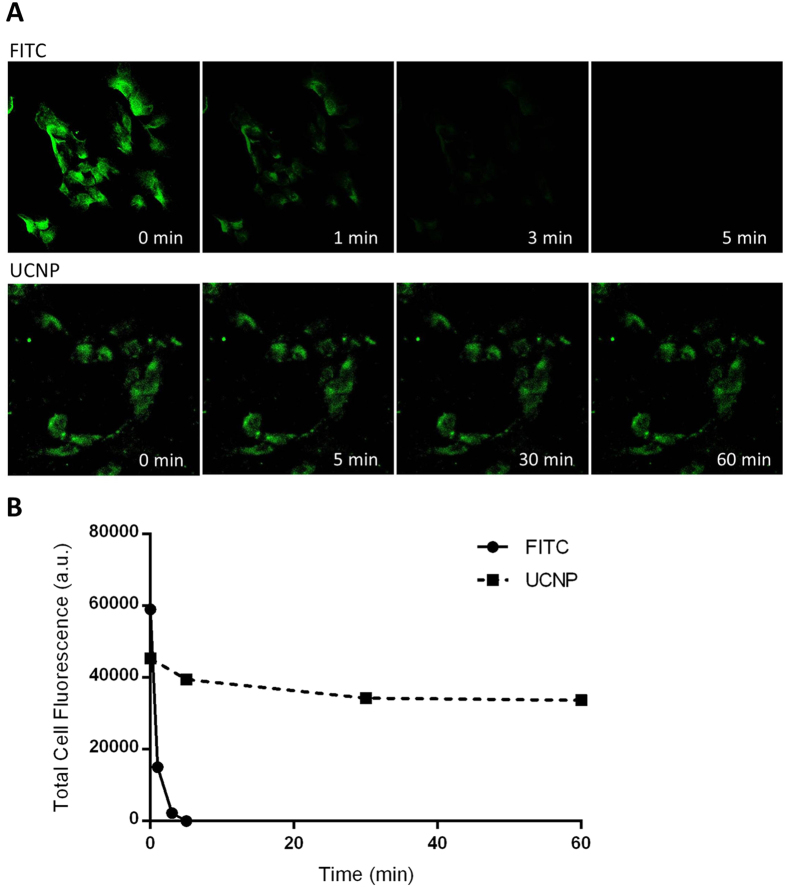
The comparison of photobleaching of fluorescence between FITC and UCNPs conjugated SA in a cell immunofluorescent assay under the excitation of 473 nm (for FITC) and 980 nm (for UCNPs), respectively, with continuous laser scanning mode. (**A**) The upper panel is the fluorescence serial imaging of FITC-SA in DU145 cells over 60 min. Green colors represent green fluorescence from FITC. The bottom panel is the fluorescence serial imaging of UINBs on DU145 cells over 60 min. Green colors represent UCL signals from UCNPs. (**B**) Comparison of cell fluorescence intensity determined by ImageJ software between FITC labeling and UCNP labeling over different scanning times.

Another advantage of UCNPs is their photostability compared to conventional fluorophores, which is also a preferred requirement for disease diagnostic detection. To investigate the optical stability of the developed MIL38-biotin-SA-PEG-UCNPs (UNIBs), we compared the UINB-labelled with MIL38-biotin-SA-FITC labelled prostate cancer cells. Both UCNPs and FITC based antibody labelled cell samples were excited under a continuous laser scanning mode at 980 nm (for UCNPs) and 473 nm (for FITC), respectively. As shown in Fig. 7A, the fluorescent signal from FITC labelled cells is very strong at the beginning, but dropped significantly one minute later and disappeared completely after three minutes under the continual excitation of the 473 nm laser. The UCNPs labelled cells showed stable fluorescent intensity maintained at the same level throughout the 60 minutes of strong NIR excitation (Fig. 7B). This data suggests that our UINBs have superior, stable optical properties, which is a promising and key feature for improved cellular disease detection and bioimaging.

## Conclusions

In summary, stable upconversion immune-nanohybrids (UINBs) have been successfully designed and developed via one-step ligand exchange strategy. The good stability of UINBs results from one-step strategy, which avoids the change of interface charge equilibration and further aggregation of UCNPs. The demonstrated potential of high-specificity prostate cancer cell diagnostic detection shown in this study benefits from the unique background-free and photostable UCNP properties together with PEG driven colloidal stability and SA-biotin driven antibody conjugation. As such, we demonstrate a versatile strategy to fabricate high-performance upconversion nanoparticles based diseased cell detection probe.

## Methods

### Materials

Unless otherwise stated, all reagents were purchased from commercial suppliers and used without further purification. Yttrium chloride hexahydrate (YCl_3_·6H_2_O, 99.99%), ytterbium chloride hexahydrate (YbCl_3_·6H_2_O, 99.98%), erbium chloride hexahydrate (ErCl_3_·6H_2_O, 99.9%), sodium hydroxide (NaOH, 98%), ammonium fluoride (NH_4_F, 99.99%), oleic acid (OA, 90%), 1-octadecene (ODE, 90%), cyclohexane (C_6_H_12_, 99.5%), ethanol (CH_3_CH_2_OH, ≥99.5%), methanol (CH_3_OH, 99.5%), dichloromethane (CH_2_Cl_2_, 99.8%), toluene (C_6_H_5_CH_3_, 99.8%), dimethylformamide (DMF, 99.8%), 1-ethyl-3-(3-dimethylaminopropyl) carbodiimide (EDC), *N*-hydroxysuccinimide (NHS), [3-(4,5-dimethylthazol-2-yl)-2,5-diphenyltetrazolium bromide] tetrazolium (MTT) and dimethyl sulfoxide (DMSO) were all purchased from Sigma–Aldrich and used as received without further purification. Pierce™ Streptavidin, EZ-Link^®^ Sulfo-NHS-LC-LC-Biotin, streptavidin-fluorescein isothiocynate (FITC) conjugate, goat anti-mouse IgG (H+L) secondary antibody, FITC conjugate and 2-(4-amidinophenyl)-1H-indole-6-carboxamidine (DAPI) were purchase from Thermo Fisher Scientific. PEG linker PO_4_-PEG_5000_-COOH was synthesized and purchased from JenKem Technology USA Inc. Monoclonal antibody MIL-38 and prostate cancer cell lines DU145 and LNCaP were all provided by Minomic Int. Ltd.

### Synthesis of OA-capped NaYF_4_: Yb^3+^/Er^3+^ Nanoparticles

Upconversion nanoparticles (OA-capped UCNPs) were synthesized using an organometallic method described previously^54^,^64^. Specifically the synthesis of NaYF_4_: 20% Yb^3+^/2% Er^3+^ is described here. Briefly, YCl_3_ (0.78 mmol), YbCl_3_ (0.18 mmol), and ErCl_3_ (0.02 mmol) were magnetically mixed with 6 mL OA and 15 mL ODE in a 100 mL three-neck round-bottom flask. The resulting mixture was heated at 160 °C under argon flow for 30 min to form a clear light yellow solution. After cooling down to 50 °C, 10 mL of methanol solution containing 0.16 g NH_4_F (4 mmol) and 0.10 g NaOH (2.5 mmol) was slowly dropped into the flask with vigorous stirring for 30 min. Then, the slurry was slowly heated and kept at 110 °C for 30 min to remove methanol and residual water. Subsequently, the reaction mixture was quickly heated up to 310 °C for 45 min and protected by an argon atmosphere. The products were isolated by adding ethanol, and centrifuged without size-selective fractionation. The final NaYF_4_: 20% Yb^3+^/2% Er^3+^ nanocrystals were re-dispersed in cyclohexane with 5 mg/mL concentration after washing with cyclohexane/ethanol several times.

### Streptavidin (SA) PEGlyation

For the PEGylation of SA, primary amines of SA were linked with PO_4_-PEG_5000_-COOH (5 kDa) using EDC/NHS chemistry. 5 mg PO_4_-PEG_5000_-COOH was dissolved in 500 μL MES buffer (0.1 M, pH 5.5) to form a 10 mg/mL solution. The solution was mixed well with 25 mg/mL EDC and 90 mg/mL NHS in MES solution and left to react for 15 min at room temperature. Ultrafiltration was performed by Vivaspin 20 (MWCO 3000) spin columns to remove excess EDC and NHS following buffer exchange into PBS (0.1 M, pH 8.0). The carboxyl activated solution was mixed with 1 mL of 2 mg/mL SA in PBS (SA: PEG molar ratio = 1:50) to react at room temperature for 2 h. Then ultrafiltration was performed by Vivaspin 500 (MWCO 50000) spin columns to remove unreacted PO_4_-PEG_5000_-COOH.

### Preparation of Streptavidin Conjugated Upconversion Nanoparticles (SA-UCNPs)

20 mg OA-capped UCNPs dispersed in 4 mL cyclohexane were mixed with a solution of NOBF_4_ (20 mg in 4 mL Dichloromethane) and was stirred at room temperature overnight. Ligand free UCNPs were obtained by centrifugation and then washed with toluene: hexane (v/v, 1/1). The prepared UCNPs were dispersed into 4 mL DMF (5 mg/mL) for long term storage. 400 μL of 5 mg/mL NOBF_4_ treated UCNPs in DMF was taken and then added to 250 μL of 2 mg/mL PEGylated SA in 1 mL PBS buffer in a 1:20 molar ratio. After adding 350 μL of 3 mg/mL PO_4_-PEG_5000_-COOH, the solution was stirred at room temperature for 24 h. The SA conjugated UCNPs were washed with PBS buffer after the reaction.

### Characterization of the UCNPs

Morphologies and sizes of UCNPs were recorded on a JEOL 2010 F Transmission Electron Microscope (TEM) operating at 200 kV. The UCNPs were sufficiently diluted so that visualization of individual particles was possible, 20 μL of the UCNPs solution was placed on a 50 Å thick carbon-coated copper grid and the excess solution was immediately removed. X-ray diffraction (XRD) analysis was carried out on a Bruker D4 X-ray diffractometer using Cu Kα radiation (λ = 0.15418 nm). The UCNPs were diluted at the same concentration at 5 mg/mL and their fluorescence emission spectra were recorded on a Fluorolog^®^-3 spectrophotometer equipped with a 980 nm VA-II diode pumped solid-state (DPSS) laser (current set at 1.50A) and a 1200 g /mm grating. The spectra were measured over a range of wavelengths from 350 nm to 850 nm. The FT-IR spectra of UCNPs with different surface functional groups were examined using a Thermo NICOLET6700 Fourier transform infrared spectrometer (FT-IR) at room temperature^65^. Dynamic Light Scattering (DLS) and zeta potential were measured to visualize size distribution and surface charge of the UCNPs by Dynamic Light Scattering Zetasizer NanoZS^66^. 1 mL of OA-capped UCNPs dispersed in cyclohexane or surface functionalized UCNPs dispersed in carbonate buffer were used for the measurement.

### MIL-38 Antibody Biotinylation

The biotinylation of antibody MIL-38 was performed using EZ-link Sulfo-NHS-LC-LC-biotin according to the manufacturer’s specifications. MIL-38 was dissolved in PBS buffer (0.1 M, pH 8) to obtain a 2 mg/mL solution. A 10 mM solution of the biotin reagent was prepared by dissolving 2.0 mg of the reagent in 300 μL of ultrapure water. 20 fold molar excess of biotin reagent was used to conjugate to the antibody by incubating at room temperature for 1 h. The excess unreacted biotin was removed by a Vivaspin 500 (MWCO 50000) ultrafiltration spin column.

## Additional Information

**How to cite this article**: Shi, Y. *et al*. Stable Upconversion Nanohybrid Particles for Specific Prostate Cancer Cell Immunodetection. *Sci. Rep*. **6**, 37533; doi: 10.1038/srep37533 (2016).

**Publisher’s note:** Springer Nature remains neutral with regard to jurisdictional claims in published maps and institutional affiliations.

## Supplementary Material

Supplementary Information
