# Supplementary material for: Stable Upconversion Nanohybrid Particles for Specific Prostate Cancer Cell Immunodetection
Source: Sci Rep. 2016 Nov 22;6:37533. doi: 10.1038/srep37533 (PMC5118722; doi:10.1038/srep37533)
Supplement: Supplementary Information [file srep37533-s1.doc]

Supplementary Information

**Stable Upconversion Nanohybrid Particles for Specific Prostate Cancer Cell Immunodetection**

**Yu Shi^1,2^, Bingyang Shi^1, 3, 4^, *, Arun V. Everest Dass^2, 4^, Yiqing Lu^4^, Nima Sayyadi^2^, Liisa Kautto^2^, Robert D. Willows^2^, Roger Chung^1, 3^, James Piper^4^, Helena Nevalainen^2^, Bradley Walsh^6^, Dayong Jin^4,5^, Nicolle H. Packer^2, 4^**

*1. International Joint Center for Biomedical Innovation, Henan University, Kaifeng, Henan, 457001, China*

*2. Department of Chemistry and Biomolecular Sciences, Macquarie University, Sydney, NSW 2109, Australia*

*3. Faculty of Medicine & Health Sciences, Macquarie University, Sydney, NSW, 2109, Australia*

*4. ARC Centre of Excellence for Nanoscale BioPhotonics, Macquarie University, Sydney, NSW, 2109, Australia.*

*5. Institute for Biomedical Materials and Devices, Faculty of Science, University of Technology Sydney, NSW, 2007, Australia.*

*6. Minomic International Ltd, Macquarie Park, Sydney, NSW, 2109, Australia.*

* Corresponding author **E-mail:** [bingyang](mailto:bingyang).shi@mq.edu.au

Contents

Cell culture and immunofluorescence assay S-2

Western blot assay S-2

MTT assay S-2

Figure S1 The SDS-PAGE electrophoresis analysis of SA-PEG mixture S-4

Figure S2 The Zeta potential of the NOBF_4_-UCNPs and SA-PEG-UCNPs S-5

Figure S3 The FT-IR absorption spectra of OA-UCNPs, NOBF_4_-UCNPs and SA-PEG-UCNPs S-6

Figure S4 The water dispersibility and stability analysis of SA-PEG-UCNPs S-7

Figure S5 The Cell Viability of SA-PEG-UCNPs on DU145 cells S-9

Figure S6 The validation of MIL-38 antibody specificity S-10

Figure S7 The negative control of UINBs cell imaging S-11

**Cell Culture and Immunofluorescence Assay.**

Prostate cancer cells (DU145, ATCC) were incubated in RPMI 1640 medium supplemented with 10 (w/v) % fetal bovine serum (FBS), streptomycine at 100 μg/mL, and penicillin at 100 U/mL. The cells were incubated at 37 ^o^C in a humidified incubator in the presence of 5% CO_2_.

Cells were seeded in a Lab-Tek® chamber slide at a density of 4×10^4^ cells per well. After cell attachment for 24 h, cells were washed with pre-warmed PBS, and fixed by 5 (w/v) % paraformaldehyde (PFA). For the FITC related IFA labelling, cells were firstly blocked by 2 (w/v) % BSA solution for 30 min, then MIL-38 antibody or biotinylated MIL-38 antibody dispersed in 2 (w/v) % BSA blocking solution was incubated with cells for 1 h. After removed excess antibody solution and washed with PBS three times, FITC conjugated SA (SA-FITC) or FITC conjugated secondary antibody (2^nd^-FITC) was added and incubated with cells for 1 h. For the upconversion immune-nanohybrids (UINBs, MIL-38-SA-PEG-UCNPs) labelling, UINBs at 100 μg/mL in PBS buffer were incubated with fixed cells for 1 h at room temperature. After incubation the cells were then washed with PBS for three times to remove excess UINBs, SA-FITC or 2^nd^-FITC and then stained with DAPI before imaging by Olympus FV1200 confocal microscope.

**Western Blot Assay**.

The DU145 and LNCaP prostate cancer cells cultured in RPMI 1640 cell culture medium over-night were collected and lysed on ice in 500 μL cell lysis buffer (1X PBS buffer, 1 (v/v) % NP40 cell lysis buffer, 0.1 (w/v) % sodium dodecyl sulphate (SDS), 5 mM ethylenediaminetetraacetic acid (EDTA), 0.5 (w/v) % sodium deoxycholate and 1 mM sodium orthovanadate) with 10 μL protease inhibitors.

Equal amounts of cell lysate (20 μL) were loaded and the proteins separated electrophoretically on 4-15 (w/v) % Bis-Tris polyacrylamide gels at 200 V for 50 min, then the proteins were transferred onto nitrocellulose membranes (NC) and blocked with 5 (w/v) % skim milk for 1 h. The membrane was incubated with monoclonal antibody MIL-38 (1:5000 dilution) and SA-FITC/2^nd^-FITC (1:10000 dilution) to detect the target protein antigen. The immunoreactive bands were visualized on an Odyssey® CLx infrared imaging system according to manufacturer’s instructions.

**MTT Assay**.

UCNP cytotoxicity was examined by [3-(4,5-dimethylthazol-2-yl)-2,5-diphenyltetrazolium bromide] te trazolium (MTT) assay on DU145 prostate cancer cells. DU145 cells were seeded in 96-well plate at a density of 4×10^4^ cells per well. The cells were incubated at 37 ^o^C in a humidified incubator in the presence of 5% CO_2_ until reach the cell confluence at 80%. DMEM cell culture medium was replaced by fresh DMEM containing SA conjugated UCNPs in different concentrations (0, 1, 2, 10, 25, 50, 100 and 200 μg/mL). After incubation at 37 ^o^C in 5% CO_2_ for 24 h and 48 h, DMEM was removed and washed by PBS three times. 100 μL of 500 μg/mL MTT solution was added to each well and incubated for 4 h. After removed the excess MTT solution, 100 μL dimethyl sulfoxide (DMSO) was added to cells to dissolve the formed formazan. The optical density of each well was measured at a wavelength of 590 nm by a microplate reader. The cell viability was calculated with following formula:


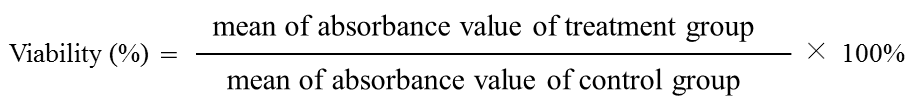


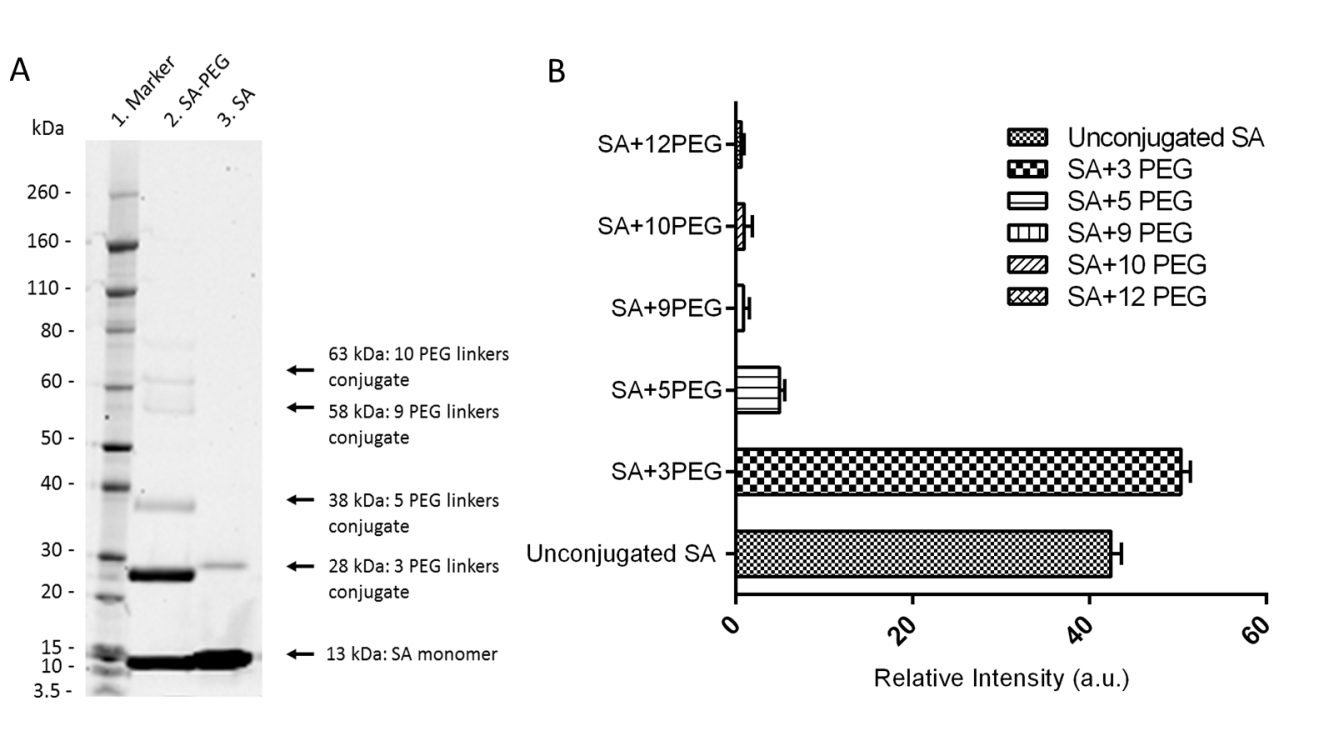


**Figure S1.** (A) SDS-PAGE analysis of SA and PEGylated SA in reducing condition and stained with coomassie brilliant blue. Lane 1: molecular mass marker. Lane 2: PEGylated SA (20 μg). Lane 3: SA (20 μg). (B) The pixel densities of bands from SA conjugated with six different amount of PEG were quantified using ImageJ software and presented as mean relative intensity. Pixel intensity of SA conjugated with 3 PEG linkers account for 50.3% of all components.


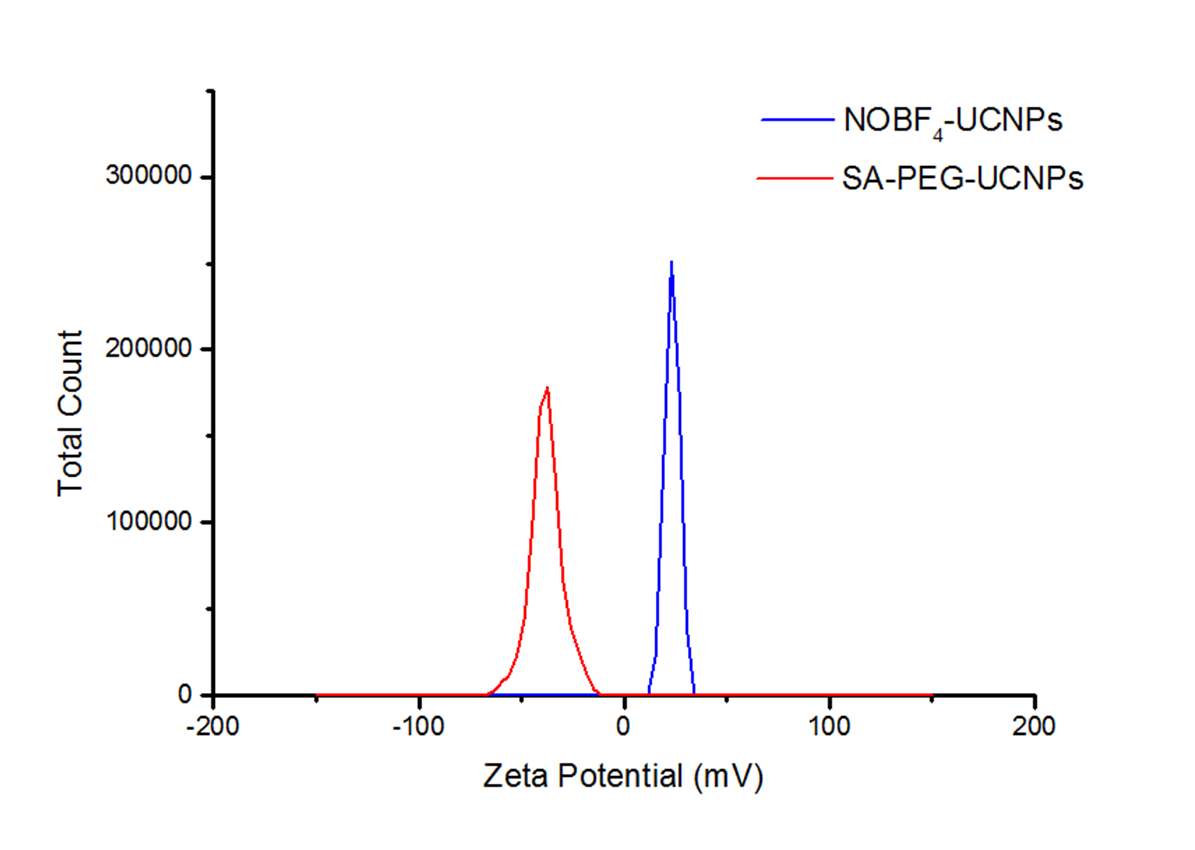


**Figure S2.** Zeta potential of the NOBF_4_-UCNPs (blue trace) dispersed in DMF and SA-PEG-UCNPs dispersed in PBS were measured at 23.3 and -36.1 mV, respectively.

**
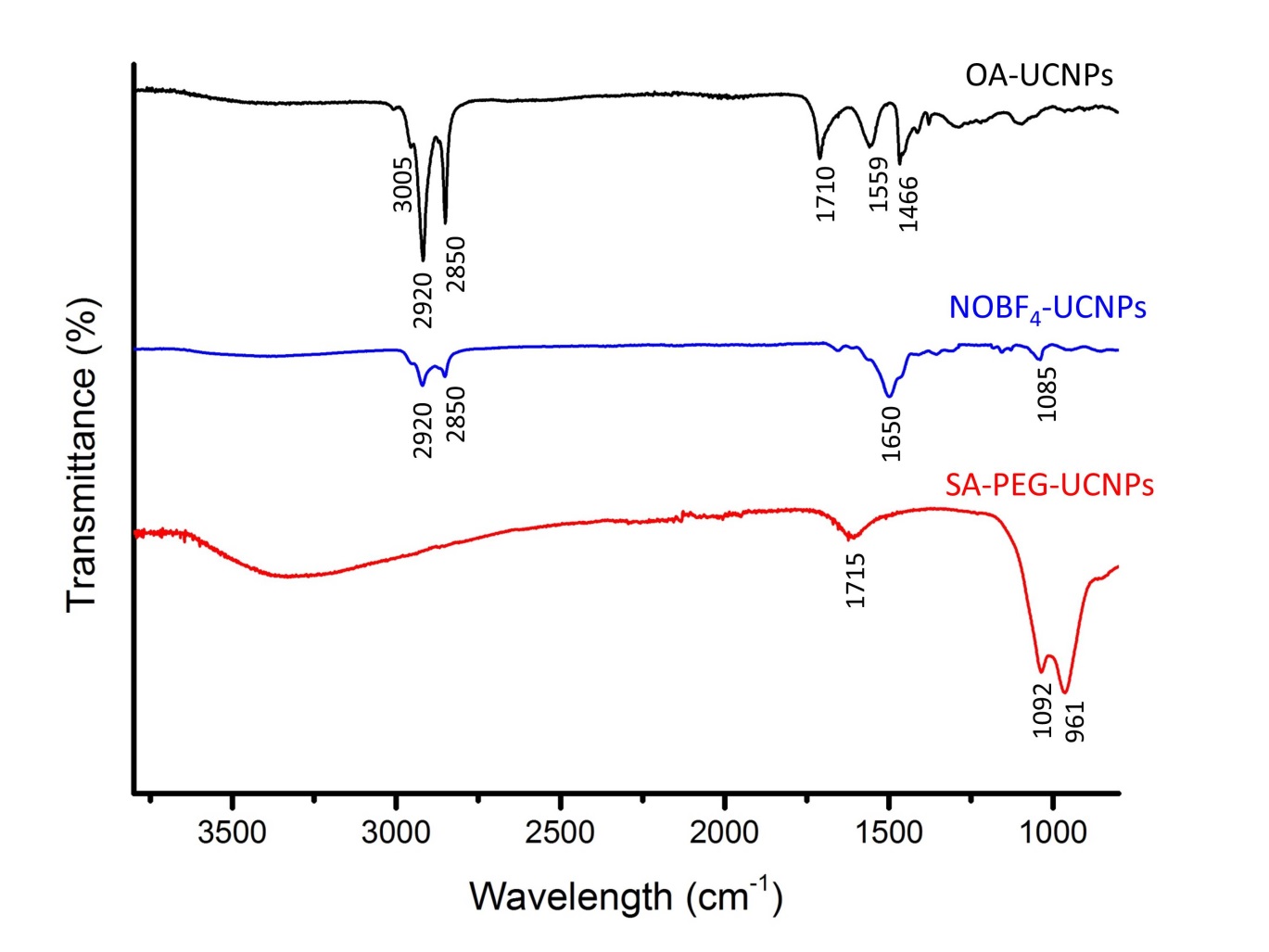
**

**Figure S3.** FT-IR absorption spectra of OA-capped UCNPs (black trace), NOBF_4_-UCNPs (blue trace) and SA-PEG-UCNPs (red trace) functionalized by NOBF_4_/SA-PO_4_-PEG_5000_-COOH.

The functional groups on the surfaces of the bare and SA-PEG-UCNPs were identified by FT-IR spectra. The surfaces of the bare UCNPs are capped with a layer of oleic acid, which acts as the surfactant and capping ligands in this synthesis. The two peaks at 2920 and 2850 cm^-1^ can be assigned to the asymmetric and symmetric stretching vibrations of methylene group, respectively, which exists in the long alkyl chain of the oleic acid molecule. Furthermore, two peaks at 1559 and 1466 cm^-1^ are associated with the asymmetric and symmetric stretching vibrations of the carboxylic group (COO), respectively. After NOBF_4_ treatment, the intensity of two characteristic peaks at 2920 and 2850 cm^-1^ are found to be greatly reduced, which suggesting the replacement of OA molecules. In comparison with OA-UCNPs, a new band at 1085 cm^-1^, which is attributed to BF_4_^-^ anions, is observed. Another new band at 1650 cm^-1^ which can be assigned to C=O stretching vibration is derived from DMF molecules as the solvent. These features suggest the replacement of OA molecules in NOBF_4_ ligand exchange. After bioconjugation, the featured bands at 2920 and 2850 cm^−1^ (corresponds to –CH_2_ group) and bands at 1457 cm^−1^ and 1563 cm^−1^ (associated with C=O) disappeared indicating the successful removal of OA. Meanwhile, the strong peak at 1092 and 961 cm^−1^ suggests the UCNPs are successfully modified with PO_4_ groups. Additionally, the strong band located around 1715 cm^−1^ corresponds to the stretching vibration of carboxyl group (C=O), confirming that the –COOH groups on the surface of SA-PEG-UCNPs.


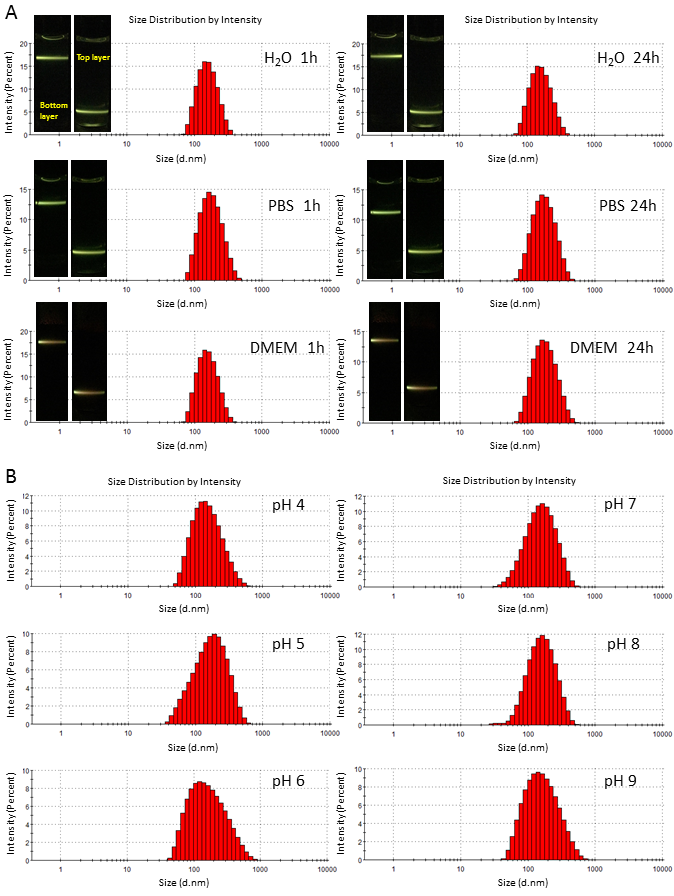


**Figure S4.** (A) The dynamic light scattering of SA-PEG-UCNPs dispersed in H_2_O, PBS buffer and DMEM cell culture medium with 10% FBS at 1 h and 24 h after their respective preparation. Left panel is luminescence photographs of SA-PEG-UCNPs dispersed in H_2_O, PBS and DMEM at 1 h and 24 h after prepared. The top layer (left side) and bottom layer (right side) of UCNP solution are excited at 980 nm laser respectively to monitor water stability. (B) The dynamic light scattering of SA-PEG-UCNPs dispersed in PBS buffer at different pH from 4.0 to 9.0 for 1 h.

**
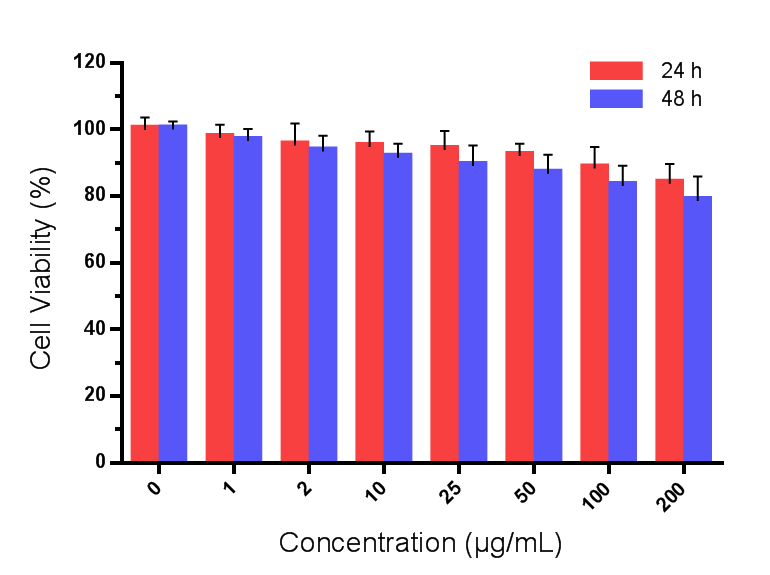
**

**Figure S5.** Cell viability of DU145 cells in the presence of SA-PEG-UCNPs with different concentrations for 24 h and 48 h at 37 °C as measured by MTT assay.


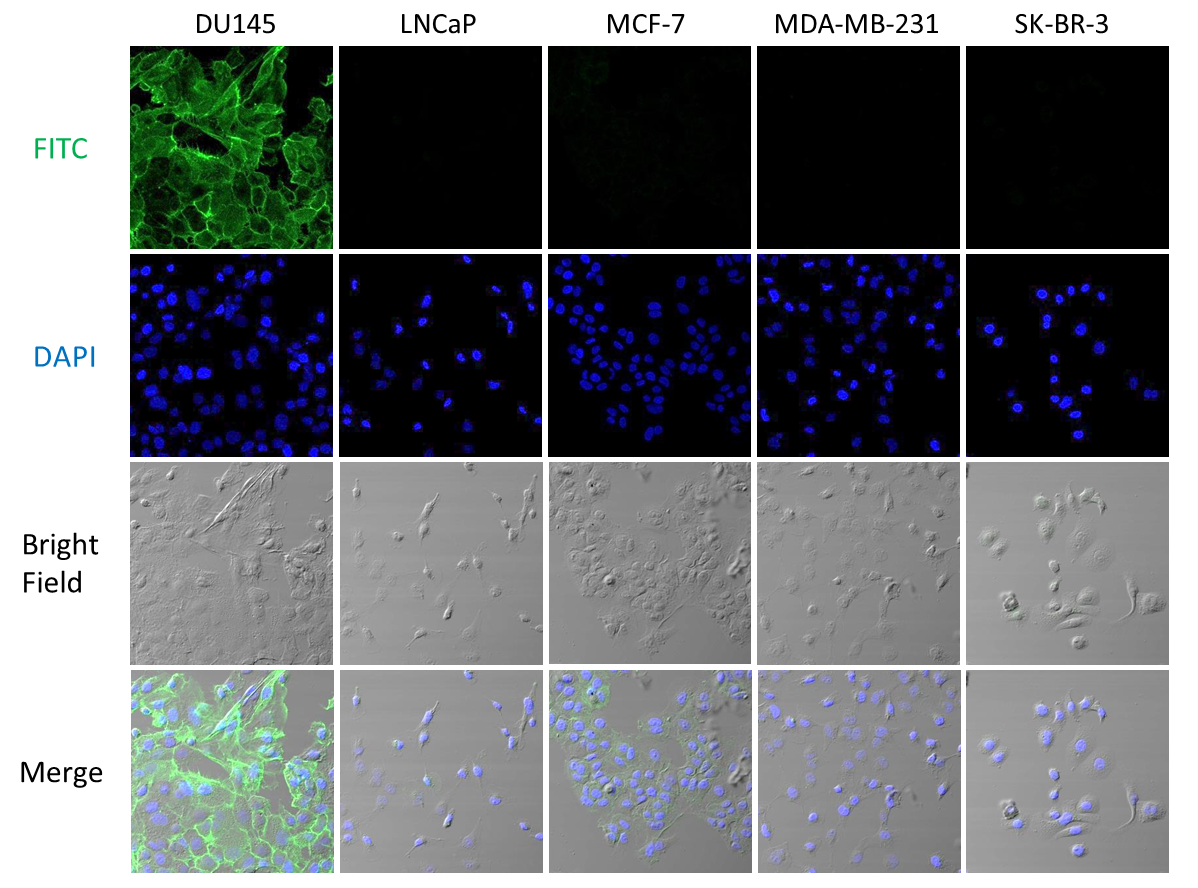


**Figure S6.** Confocal luminescence imaging of the FITC-conjugated antibody MIL-38 incubated with prostate cancer cell lines DU145 and LNCaP and breast cancer cell lines MCF-7, MDA-MB-231 and SK-BR-3. Green and blue colors represent green and blue fluorescence from FITC and DAPI, respectively.


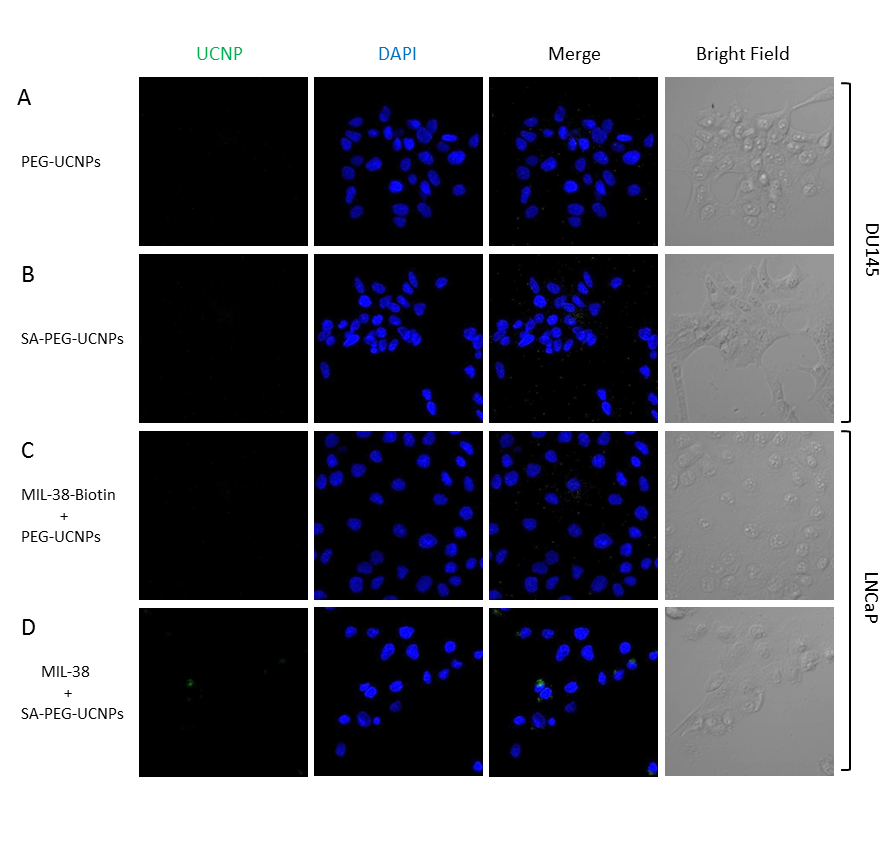


**Figure S7.** Control experiment of confocal upconversion fluorescence imaging of (A) PEG-UCNPs and (B) SA-PEG-UCNPs incubated with DU145 prostate cancer cells. (C) PEG-UCNPs labeled with MIL-38-Biotin and (D) SA-PEG-UCNPs labeled with MIL-38 on LNCaP prostate cancer cells. All images were taken under the identical instrumental condition and presented at the same intensity scale. Green and blue colors represent UCL signals and blue fluorescence from UCNPs and DAPI, respectively.
